# Supplementary material for: Complexome profiling of the Chlamydomonas psb28 mutant reveals TEF5 as an early PSII assembly factor
Source: Plant Cell. 2025 Mar 17;37(6):koaf055. doi: 10.1093/plcell/koaf055 (PMC12164590; doi:10.1093/plcell/koaf055)
Supplement: koaf055_Supplementary_Data [file koaf055_supplementary_data.zip › psb28_interactive.zip!/plots/intensity/qualityPlots/peptides/_Cre11.g467769.t1.1.html]

### Cre11.g467769.t1.1

Trivial name:   
SeqCoverage [median]: 35.50  
Mass: 18.31 kDa  
  
 

| Sequence | MissedCleavage | Mass [Da] | wtIntensity | muIntensity | Score |
| --- | --- | --- | --- | --- | --- |
| APNNAPR | 0 | 738.38 | 0.00 | 517.19 | 102.81 |
| FGGSPPPPPPPPPQPTR | 0 | 1721.89 | 9569.22 | 16488.78 | 142.97 |
| GLADQADAVSALEGLK | 0 | 1556.80 | 10466.58 | 40125.19 | 306.21 |
| QVDDALGDLIR | 0 | 1213.63 | 9086.97 | 28379.61 | 174.98 |
| TSFQPPTR | 0 | 932.47 | 1191.23 | 3710.52 | 122.52 |
| TSFQPPTRR | 1 | 1088.57 | 3366.46 | 13041.95 | 90.15 |
|  |  |  | 33680.5 | 102263.2 |  |
